# Designer genetically encoded voltage‐dependent calcium channel inhibitors inspired by RGK GTPases

**DOI:** 10.1113/JP276544

**Published:** 2020-04-21

**Authors:** Henry M. Colecraft

**Affiliations:** ^1^ Department of Physiology and Cellular Biophysics Department of Pharmacology and Molecular Signaling Columbia University Vagelos College of Physicians and Surgeons New York NY USA

**Keywords:** calcium channel, ion channel regulation, nanobody, RGK, ubiquitin

## Abstract

High‐voltage‐activated calcium (Ca_V_1/Ca_V_2) channels translate action potentials into Ca^2+^ influx in excitable cells to control essential biological processes that include; muscle contraction, synaptic transmission, hormone secretion and activity‐dependent regulation of gene expression. Modulation of Ca_V_1/Ca_V_2 channel activity is a powerful mechanism to regulate physiology, and there are a host of intracellular signalling molecules that tune different aspects of Ca_V_ channel trafficking and gating for this purpose. Beyond normal physiological regulation, the diverse Ca_V_ channel modulatory mechanisms may potentially be co‐opted or interfered with for therapeutic benefits. Ca_V_1/Ca_V_2 channels are potently inhibited by a four‐member sub‐family of Ras‐like GTPases known as RGK (Rad, Rem, Rem2, Gem/Kir) proteins. Understanding the mechanisms by which RGK proteins inhibit Ca_V_1/Ca_V_2 channels has led to the development of novel genetically encoded Ca_V_ channel blockers with unique properties; including, chemo‐ and optogenetic control of channel activity, and blocking channels either on the basis of their subcellular localization or by targeting an auxiliary subunit. These genetically encoded Ca_V_ channel inhibitors have outstanding utility as enabling research tools and potential therapeutics.

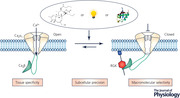

## Graphical abstract

RGK proteins are small Ras‐like GTPases that potently inhibit voltage‐gated calcium (CaV) channels by binding their auxiliary b subunits. Mechanistic insights into how RGK proteins inhibit CaV channels has been exploited to develop novel genetically‐encoded CaV channel inhibitors that can be acutely activated by small molecules or light, or produce constitutive inhibition via targeted ubiquitination using CaVb‐binding nanobodies. Advantages of such genetically‐encoded CaV channel inhibitors include their ability to be selectively targeted to specific tissue, cell types, sub‐cellular localization, and distinct CaV channel macromolecular complexes.

## Voltage‐gated calcium channels: basic structure, function and regulation

Ca^2+^ is a universal second messenger that regulates numerous biological functions in virtually all cells (Berridge *et al*. 2000). Cytoplasmic Ca^2+^ in cells is kept low (100 nM) but rises in response to diverse stimuli (to ∼1 μM) to initiate functional responses through the action of a variety of Ca^2+^‐dependent proteins. The source of signalling Ca^2+^ is from either intracellular stores or the extracellular milieu. There are a variety of integral membrane proteins on the plasma membranes of diverse cell types that permit the entry of Ca^2+^ in response to specific stimuli. Amongst these are the family of voltage‐dependent Ca^2+^ channels (VDCCs) which gate Ca^2+^ entry into cells in response to changes in membrane potential. VDCCs are sub‐divided into two categories based on the threshold voltage for activation – low‐voltage‐activated (LVA) and high‐voltage‐activated (HVA) Ca^2+^ channels, respectively. There are three distinct LVA (Ca_V_3.1 – Ca_V_3.3) and seven HVA (Ca_V_1.1 – Ca_V_1.4; Ca_V_2.1 – Ca_V_2.3) (Catterall, 2011; Zamponi *et al*. 2015). VDCCs play many essential roles in the biology of excitable cells. As examples, Ca^2+^ influx through VDCCs: contributes to pacemaking in many cell types including the sino‐atrial node of the heart and substantia nigra (Ca_V_3; Ca_V_1.3) (Guzman *et al*. 2010; Mesirca *et al*. 2015); regulates neuronal excitability by coupling to Ca^2+^‐activated K^+^ channels (Ca_V_1.2; Ca_V_2.1; Ca_V_2.2) (Marrion & Tavalin, 1998; Womack *et al*. 2004); controls the heartbeat by coupling electrical excitation to muscle contraction in cardiomyocytes (Ca_V_1.2) (Bers, 2002); enables communication among neurons by triggering presynaptic neurotransmitter release (Ca_V_2.1‐Ca_V_2.3) (Sudhof, 2012); promotes the release of hormones, e.g. insulin, adrenaline (epinephrine), essential for metabolic and physiological homeostasis (Ca_V_1.2, Ca_V_1.3, Ca_V_2) (Braun *et al*. 2008); and engenders long‐term changes in cellular function by regulating gene expression (Ca_V_1.2, Ca_V_1.3) (Wheeler *et al*. 2012).

Functional HVA Ca^2+^ channels *in vivo* are multi‐subunit complexes comprising distinct pore‐forming α_1_ subunits (α_1A_ – Ca_V_2.1; α_1B_ – Ca_V_2.2; α_1C_ – Ca_V_1.2; α_1D_ – Ca_V_1.3; α_1E_ – Ca_V_2.3; α_1F_ – Ca_V_1.4; and α_1S_ – Ca_V_1.1) assembled with calmodulin and auxiliary β (Ca_V_β_1_ – Ca_V_β_4_), α_2_δ (α_2_δ‐1 – α_2_δ‐3), and γ subunits (Zamponi *et al*. 2015). In heterologous expression studies, co‐expression with Ca_V_β is necessary for efficient α_1_‐subunit trafficking to the plasma membrane (Buraei & Yang, 2010). Consistent with an essential *in vivo* role, β_1_‐null mice die at birth due to asphyxiation (Gregg *et al*. 1996) and β_2_ knock‐out is embryonic lethal due to cardiac defects (Weissgerber *et al*. 2006). Nevertheless, recent *in vivo* data in adult cardiomyocytes indicate an exception to the absolute necessity for Ca_V_β to enable trafficking of α_1C_ to the surface membrane of adult heart cells. Cardiac‐specific excision of Ca_V_β_2_, the dominant Ca_V_β isoform in heart, reduced Ca_V_β_2_ protein by 96% while decreasing Ca_V_1.2 current amplitude by only 26% (Meissner *et al*. 2011). Further, a transgenic mouse expressing a dihydropyridine‐resistant α_1C_ mutant that does not bind Ca_V_β displayed ample DHP‐resistant Ca_V_1.2 current, indicating a robust Ca_V_β‐independent trafficking to the sarcolemma (Yang *et al*. 2019). It remains to be determined whether and to what extent Ca_V_β‐independent trafficking happens in other cell types and other Ca_V_1/Ca_V_2 isoforms at different developmental stages. Beyond their impact on Ca_V_1/Ca_V_2 trafficking, Ca_V_β isoforms alter multiple channel gating properties – shift the voltage dependence of channel activation to the left, increase single channel open probability, impart distinctive rates of inactivation, and endow different steady‐state inactivation profiles (Buraei & Yang, 2010). α_2_δ subunits promote surface trafficking and can alter biophysical properties of particular Ca_V_1/Ca_V_2 channels (Dolphin, 2012). γ subunits are associated with Ca_V_1.1 channels (Kang & Campbell, 2003; Wu *et al*. 2016); their association with other Ca_V_1/Ca_V_2 channels *in vivo* is unclear. Multiple CaM binding sites have been described at different locations in distinct Ca_V_1/Ca_V_2 channels (Van Petegem *et al*. 2005; Dick *et al*. 2008; Mori *et al*. 2008; Ben‐Johny & Yue, 2014). CaM binds to the C‐terminus of most Ca_V_1/Ca_V_2 channels in a fairly conserved region containing an IQ motif (Erickson *et al*. 2001; Kim *et al*. 2008, 2004, 2010; Mori *et al*. 2008). Binding of apoCaM to this region has been shown to enhance the open probability, *P*_o_, of Ca_V_1.3 channels (Adams *et al*. 2014). Cryo electron microscopy structures of Ca_V_1.1 and Ca_V_3.1 channels have yielded invaluable insights into Ca_V_ channel structure, three‐dimensional assembly and modulation by ligands (Wu *et al*. 2016; Zhao *et al*. 2019 *a*,*b*).

An important feature of HVA Ca_V_ channels is that their activity is not static but is dynamically regulated both by stably associated proteins as well as transiently interacting signalling molecules. Typically, these regulatory mechanisms have profound physiological importance; their dysregulation can cause pathology, and they can be co‐opted or interfered with for therapy. Examples of these regulatory mechanisms include: Ca^2+^‐dependent inactivation of Ca_V_1.2 channels mediated by preassociated CaM, a negative feedback mechanism which when disrupted leads to prolonged cardiac action potentials and life‐threatening cardiac arrhythmias (Peterson *et al*. 1999; Zuhlke *et al*. 1999; Alseikhan *et al*. 2002); protein kinase A mediated up‐regulation of cardiac Ca_V_1.2, essential for the physiologically critical fight‐or‐flight response (Kamp & Hell, 2000); voltage‐dependent inhibition of Ca_V_2 channels by G_βγ_ subunits (Dolphin, 2003), a mechanism for tuning synaptic strength that is important for the analgesic effects of opiates.

## RGK GTPase inhibition of Ca_V_ channels: discovery and mechanisms

The seminal report of the functional interaction between RGK proteins and Ca_V_1/Ca_V_2 channels was in 2001 – a yeast two‐hybrid screen of MIN6 cells using Ca_V_β_3_ as bait fished out Gem/Kir as an interacting protein (Beguin *et al*. 2001). Co‐expressing Gem with recombinant Ca_V_1.3 or Ca_V_1.2 in *Xenopus* oocytes resulted in a marked inhibition of calcium channel current. Gem was initially discovered as a mitogen‐induced gene in human T cells (Maguire *et al*. 1994) and belongs to a sub‐family of Ras‐like monomeric G‐proteins with three other members: Rad (Ras associated with diabetes), originally discovered as a protein over‐expressed in skeletal muscle of diabetic patients (Reynet & Kahn, 1993); Rem, first identified using a degenerate cloning strategy based on homology to Gem and Rad (Finlin & Andres, 1997); and Rem 2, cloned from a rat brain cDNA library (Finlin *et al*. 2000). Subsequent to the original report of Gem inhibition of Ca_V_1.2 and Ca_V_1.3, it was shown that this phenomenon also extended to Rad and Rem, which both potently inhibited Ca_V_1.2 channels (Finlin *et al*. 2003), and Rem 2 (Chen *et al*. 2005; Finlin *et al*. 2005). Over‐expressing any RGK protein markedly suppresses endogenous Ca_V_1/Ca_V_2 channels in native cells including cardiac myocytes, neurons and skeletal muscle (Murata *et al*. 2004; Chen *et al*. 2005; Bannister *et al*. 2008; Wang *et al*. 2010; Xu *et al*. 2010; Puckerin *et al*. 2018). A recent elegant study revealed that endogenous Rad in cardiomyocytes constitutively exerts a gating brake on a fraction of Ca_V_1.2 channels. This inhibition is relieved by protein kinase A phosphorylation of Rad, and is the long sought‐after mechanism by which β‐adrenergic agonists increase cardiac Ca_V_1.2 to enhance inotropy during the fight‐or‐flight response (Liu *et al*. 2020).

**Figure 1:**
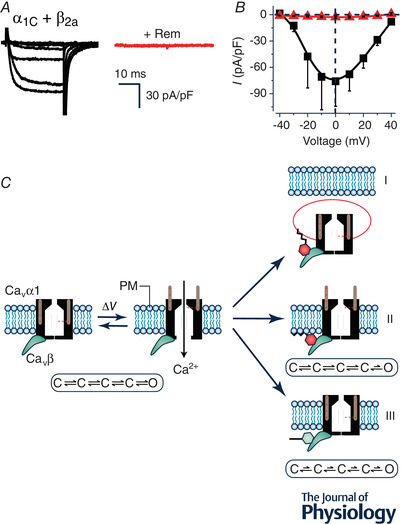
Rem inhibition of reconstituted Ca_V_1.2 channels *A*, exemplar family of whole‐cell Ba^2+^ currents from recombinant Ca_V_1.2 channels (α_1C _+ β_2a_) reconstituted in HEK293 cells either without (left) or with (right) co‐expression of Rem. *B*, population *I‐V* curves from Ca_V_1.2 channels in the absence (■) or presence (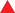) of co‐expressed Rem. *C*, schematic diagram showing three distinct mechanisms (I‐III) utilized by Rem to inhibit recombinant Ca_V_1.2 channels. In mechanism I, co‐expressed Rem results in a decrease in the number of channels at the cell surface (N) due to enhanced Ca_V_1.2 endocytosis. Mechanism II involves a reduction in the open probability (*P*_o_) of channels residing on the plasma membrane without impacting on voltage sensor movement as measured by total gating charge (*Q*_max_). This mechanism requires Rem simultaneously binding to the Ca_V_β subunit (using the guanine nucleotide binding domain) and the plasma membrane (via the polybasic distal C‐terminus). Mechanism III involves an impaired movement of the voltage sensor movement of surface channels as measured by a decreased *Q*_max_ (observed even when *N* is completely rescued by co‐expressing dominant negative dynamin). Mechanism III is blocked by a mutation (T94N) that favours GDP over GTP binding to Rem, suggesting it requires GTP‐bound Rem.

How do RGK proteins inhibit Ca_V_1/Ca_V_2 channels? The answer to this seemingly simple question turned out to be surprisingly complex. The whole‐cell current (*I*) is related to microscopic channel properties by the relation *I* = *F*_A_ × *N *× *i *×* P*_o_; where *F*_A_ is the fraction of activatable channels, *N* is the total number of channels, *i* is the unitary current amplitude, and *P*_o_ is the open probability. In principle, RGK proteins could inhibit *I* by reducing any of the four parameters or a combination of them. We found that Rem inhibits Ca_V_1.2 channels reconstituted in HEK293 cells in at least three distinct ways (Fig. 1) (Yang *et al*. 2010). First, in this system, Rem reduced Ca_V_1.2 surface density (*N*) by 65%, an effect that was reversed by co‐expressing dominant negative dynamin. The second mechanism involved a reduction in channel *P*_o_, which occurred without an impact on Ca_V_1.2 voltage sensor movement, suggesting an impairment in coupling between voltage sensors and opening of the channel pore. This mechanism specifically required simultaneous association of Rem with the plasma membrane (mediated by a polybasic distal C‐terminus) and Ca_V_β in the channel complex (via the guanine nucleotide binding domain). Finally, a third mechanism entailed a reduction in Ca_V_1.2 maximal gating charge (*Q*_max_) that was not accounted for by a change in channel surface density, suggesting an immobilization of one or more voltage sensors. This third mechanism required GTP bound to Rem and would have the practical effect of diminishing both *F*_A_ and *P*_o_. While these three mechanisms of Rem inhibition of Ca_V_1.2 can be observed in HEK293 cells, their relative prevalence may differ in other cell types. For example, over‐expression of Rem in cardiac myocytes markedly depresses Ca_V_1.2 whole‐cell current without an apparent change in channel surface density as indicated by immunofluorescence, and the acute rescue of near‐maximal current with BAYK 8644 (Xu *et al*. 2010).

From a macroscopic perspective all four RGKs profoundly inhibit all Ca_V_1/Ca_V_2 channels when over‐expressed. Nevertheless, underneath this apparent uniformity, there are important distinctions in the mechanisms of inhibition that extend to both the different RGKs as well as to individual channel types (Yang & Colecraft, 2013). Rem2 was found to inhibit Ca_V_1.2 channels in mouse insulinoma MIN6 cells (Finlin *et al*. 2005) and also Ca_V_2.2 channels in tsA201 cells without reducing the number of channels at the cell surface (Chen *et al*. 2005). By reconstituting channels with either wild type Ca_V_β or a mutant Ca_V_β that loses binding to RGK proteins, we found that Rem and Rad could inhibit Ca_V_1.2 and Ca_V_2.2 (but not the other Ca_V_1/Ca_V_2 channel types) using either β‐binding‐dependent or β‐binding‐independent mechanisms (Yang *et al*. 2012; Puckerin *et al*. 2018, 2016). In the particular case of Rem inhibition of Ca_V_1.2, the β‐binding‐independent mechanism of inhibition is mediated by an interaction of the Rem distal C‐terminus with the α_1C_ N‐terminus region just upstream of the first transmembrane spanning segment of the channel (Yang *et al*. 2012). By contrast, Gem and Rem2 utilize solely a β‐binding‐dependent mechanism to inhibit Ca_V_1/Ca_V_2 channels. Overall, insights into the mechanisms and physical determinants of RGK inhibition of Ca_V_1/Ca_V_2 channels has proven invaluable to the broad objective of drawing inspiration from these proteins as prototype molecules to design next‐generation genetically encoded Ca_V_ channel inhibitors as research tools and potential therapeutics.

## RGK‐inspired genetically encoded Ca_V_ channel inhibitors

Blocking Ca_V_1/Ca_V_2 channels with small molecules or toxins is a prevailing or prospective therapeutic strategy for many serious diseases including hypertension, chronic pain, cardiac arrhythmias, Parkinson's disease and stroke (Zamponi *et al*. 2015; Zamponi, 2016). While convenient, small molecule Ca_V_ channel blockers have limitations, some of which may be circumvented by genetically encoded inhibitors (Xu & Colecraft, 2009). First, they lack tissue specificity since small molecules are typically widely distributed in the body after administration, and distinct VDCCs are present across many different tissues, organs and cell types. Second, VDCCs show an immense molecular and functional diversity stemming from their organization into distinct macromolecular complexes, and sub‐cellular localizations that are poorly discriminated by small molecules. These two gap areas could potentially be filled by novel genetically encoded Ca_V_ channel inhibitors designed to target molecularly distinct VDCC macromolecular complexes in a tissue‐ or cell‐specific manner. While RGK proteins themselves are potent VDCC inhibitors, their usefulness as research tools or therapeutics is limited by several factors: (1) they are non‐selective, as they indiscriminately inhibit all Ca_V_1/Ca_V_2 channel types; (2) they are constitutive inhibitors, thus providing poor temporal and spatial control of channel block; and (3) they are non‐specific as they interact with and regulate other proteins such as enzymes and the cytoskeleton in cells (Yang & Colecraft, 2013). Over the last few years, using RGK proteins themselves as inspiration, we and others have explored different ways to engineer new genetically encoded Ca_V_ channel inhibitors that improve on various aspects of functional Ca_V_ channel block that are lacking in wild‐type RGK proteins.

Our finding that Rem specifically inhibits Ca_V_1.2 using both a β‐binding‐dependent and α_1C_‐binding‐dependent mechanism but used only a β‐binding‐dependent mechanism to block other Ca_V_1/Ca_V_2 channel types suggested a simple method to create a Ca_V_1.2‐selective genetically encoded Ca_V_ channel inhibitor – introduce mutations in Rem that weaken its interaction with Ca_V_β without altering the tertiary structure of the protein. Indeed, such mutations (Rem[R200A/L227A]) were identified by an extensive mutagenesis study (Beguin *et al*. 2007). Consistent with the hypothesis, Rem[R200A/L227A] selectively inhibited Ca_V_1.2, but not other Ca_V_1/Ca_V_2 channels, reconstituted in HEK293 cells (Puckerin *et al*. 2018). The ability of Rem[R200A/L227A] to discriminate between Ca_V_1.2 and Ca_V_1.3 was especially notable given the difficulty of identifying small molecules that can effectively distinguish between these two L‐type channel subtypes (Zamponi *et al*. 2015). Using a similar logic, we found that Rad[R208A/L235A] selectively blocked Ca_V_1.2 and Ca_V_2.2, consistent with the finding that Rad inhibits these two channels using both β‐binding‐dependent and β‐binding‐independent mechanisms (Puckerin *et al*. 2018). Importantly, both Rem[R200A/L227A] and Rad[R208A/L235A] strongly inhibited Ca_V_1.2 channels in cardiomyocytes, indicating that the β‐binding‐independent mechanism of inhibition is operational in this native environment. Similarly, the two proteins inhibited HVA Ca_V_ channel currents in dorsal root ganglion (DRG) neurons to different extents, reflecting their varying selectivity for Ca_V_1.2 and Ca_V_1.2/Ca_V_2.2 channels, respectively (Puckerin *et al*. 2018).

**Figure 2:**
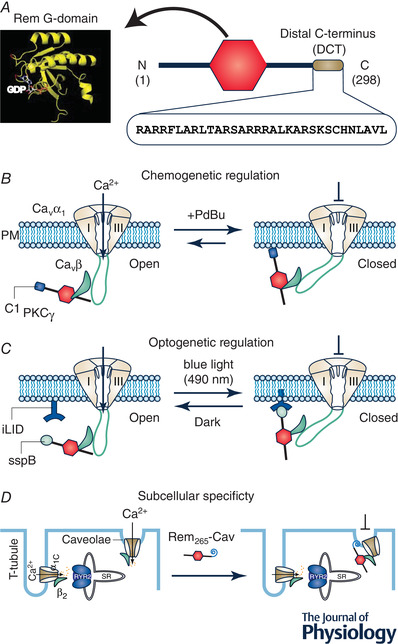
Replacing Rem distal C‐terminus for novel spatio‐temporal control of Ca_V_ channel inhibition *A*, Rem structure consists of a guanine nucleotide binding domain (G‐domain) flanked by N‐ and C‐termini. The Rem distal C‐terminus (DCT), comprising the last 32 residues of the protein, is a polybasic peptide that mediates binding to the plasma membrane and is necessary for Ca_V_ channel inhibition. *B*, replacing Rem DCT with C1 domain from protein kinase C γ (C1_PKCγ_) enables acute recruitment of the engineered Rem to the plasma membrane with a small molecule phorbol ester, PdBu. Co‐expressed Ca_V_1/Ca_V_2 channels are inhibited concomitantly with Rem_265_‐C1_PKCγ_ association with the plasma membrane. This chemogenetic configuration provides acute temporal control over Ca_V_ channel inhibition that is slowly reversible. *C*, optogenetic control of Rem inhibition of Ca_V_ channels was achieved using the photodimerizer pair, iLID (LOV2‐ssrA) and sspB. The Rem DCT was replaced with sspB via varying linkers (creating optoRGK) while iLID was constitutively anchored to the plasma membrane. Exposure of cells to blue light (470 nm) enabled acute recruitment of optoRGK to the plasma membrane and inhibition of Ca_V_1.2 channels. Both plasma membrane association of optoRGK and Ca_V_1.2 channel inhibition were reversed in the dark. *D*, replacing Rem DCT with a caveolae‐targeting peptide enabled selective inhibition of caveolae‐targeted Ca_V_1.2 channels in cardiomyocytes while sparing dyadic Ca_V_1.2 channels that mediate cardiac excitation‐contraction coupling.

Rem associates with the plasma membrane via the 32‐residue distal C‐terminus (DCT) using hydrophobic and electrostatic interactions. Deletion of the DCT abolishes both Rem membrane targeting and inhibition of Ca_V_1/Ca_V_2 channels (Finlin *et al*. 2003; Yang *et al*. 2007). The requirement for Rem binding to the plasma membrane for Ca_V_ channel inhibition has been exploited to engineer Rem derivatives that enable chemo‐ and optogenetic control of channel inhibition, and also subcellular specificity (Fig. 2). We replaced Rem DCT with the C1 domain from protein kinase γ, creating Rem_1‐265_‐C1_PKCγ_ which when expressed in cells was primarily distributed in the cytosol but could be rapidly recruited to the plasma membrane with a small molecule, phorbol‐12,13‐dibuytrate (PdBu). The PdBu‐induced recruitment of Rem_1‐265_‐C1_PKCγ_ caused a concomitant rapid inhibition of Ca_V_1/Ca_V_2 channel currents (Fig. 2*B*) (Yang *et al*. 2013, 2007). The generality of this chemogenetic regulation was demonstrated by development of a FK506‐binding protein (FKBP)‐tagged Rem_265_ version that could be recruited to the membrane to inhibit Ca_V_1/Ca_V_2 channels using rapamycin‐mediated heterodimerization in cells that also expressed constitutively membrane‐targeted FRB (a fragment of mTOR) (Crabtree & Schreiber, 1996; Inoue *et al*. 2005; Yang *et al*. 2007). Similarly, a 490 nm blue light‐mediated heterodimerization strategy was utilized to develop optogenetic control of Rem inhibition (Fig. 2*C*). The approach is based on a light‐induced protein‐protein interaction created by inserting a bacterial peptide, ssrA, into a naturally occurring photoswitch, light‐oxygen‐voltage 2 (LOV2) domain from *Avena sativa* (Guntas *et al*. 2015). In the dark, SsrA is sterically obstructed from interacting with a binding partner, sspB. With blue light, this steric inhibition is relieved, allowing SsrA to bind SspB. Extensive bioengineering of LOV2‐SsrA yielded an improved light inducible dimer (iLID) in which the affinity of the photoswitch for SspB changes > 50‐fold with light illumination (Guntas *et al*. 2015). Ma *et al*. (2018) replaced Rem DCT with SspB (creating optoRGK) and anchored iLID constitutively to the plasma membrane using Lyn11, a plasma membrane‐tethering peptide from the tyrosine protein kinase, Lyn. Exposure of cells to blue light led to rapid recruitment of optoRGK to the plasma membrane and resulted in Ca_V_ channel inhibition that was quickly reversed in the dark (Ma *et al*. 2018) (Fig. 2*C*). Finally, as a demonstration of inhibiting Ca_V_ channels with subcellular specificity, replacing the Rem C‐terminus with a caveolin‐targeting peptide enabled selective inhibition of caveolae‐localized Ca_V_1.2 in cardiac myocytes, without significantly affecting non‐caveolae Ca_V_1.2 channels responsible for excitation‐contraction coupling (Fig. 2*D*) (Makarewich *et al*. 2012).

The next conceptual advance came from further consideration of why Rem inhibition of Ca_V_1.2 *P*_o_ had the dual requirement for Ca_V_β binding and plasma membrane association? We hypothesized that Rem binding to the plasma membrane ‘pulled’ on the I‐II loop via the associated Ca_V_β subunit and induced a conformation of the channel with a low *P*_o_. This hypothesis led to a testable prediction that we could potentially evoke a similar low‐*P*_o_ channel conformational state by directly attaching a membrane‐targeting module to auxiliary Ca_V_β subunits, thereby bypassing the need for an RGK altogether (Yang *et al*. 2013). To accomplish this, we fused the C1_PKCγ_ onto the C‐terminus of Ca_V_β_3_ (generating β_3_‐C1_PKCγ_) which enabled a PdBu‐induced association of β_3_ with the plasma membrane (Yang *et al*. 2013). Channels reconstituted with β_3_‐C1_PKCγ_ yielded robust baseline whole‐cell currents that were inhibited by exposure to PdBu. The kinetics and extent of inhibition could be tuned by serial truncations of the disordered β_3_ C‐terminus (shortening the β_3_ C‐terminus sped up the onset and deepened the extent of inhibition) (Yang *et al*. 2013). While this result was in accord with the stated hypothesis, it was, nevertheless surprising, because β_2a_ and β_2e_ subunits are naturally membrane‐associated via their N‐termini (Chien *et al*. 1998; Takahashi *et al*. 2003). β_2a_ is palmitoylated, while the N‐terminus of β_2e_ forms a helix that associates with the plasma membrane using electrostatic and hydrophobic interactions (Miranda‐Laferte *et al*. 2014). However, neither β_2a_ nor β_2e_ constitutively inhibit channels (rather, they both slow down voltage‐dependent inactivation of Ca_V_1/Ca_V_2 channels) (Takahashi *et al*. 2003). An apparent explanation for this discrepancy arose from the finding that placing the C1_PKCγ_ module on the β_3_ N‐terminus yielded a construct that did not effectively inhibit Ca_V_ channels in response to PdBu, indicating that the phenomenon is sensitive to the polarity of the membrane‐targeting module on Ca_V_β (Yang *et al*. 2013). This suggests a geometric constraint to this mode of inhibition. Based on these results, we probed whether other cytosolic proteins that bound other intracellular loops of Ca_V_ channels could be transformed into Ca_V_1/Ca_V_2 inhibitors simply by introducing a membrane binding module to them. Indeed, we found that 14‐3‐3, a protein previously reported to bind to Ca_V_2.2 C‐terminus (Li *et al*. 2006), could be turned into either a PdBu‐inducible or constitutive inhibitor by attaching C1_PKCγ_ or a palmitoylated peptide, respectively (Yang *et al*. 2013). Unexpectedly, we found that 14‐3‐3‐C1_PKCγ_ also effectively inhibited Ca_V_1.2 and Ca_V_2.1 channels in a phorbol ester‐dependent manner, revealing that these other channels also interacted with 14‐3‐3. We termed this general mechanism ChIMP, an acronym for ‘channel inactivation by membrane‐tethering an associated protein’ (Yang *et al*. 2013). Beyond Ca_V_1/Ca_V_2 channels, ChIMP may also be used either as an investigational tool or method to develop genetically encoded modulators for other ion channels. In this regard, we exploited ChIMP to reveal that calmodulin is preassociated with TMEM16A and TMEM16B Ca^2+^‐activated chloride channels and mediates Ca^2+^‐dependent sensitization of activation as well as Ca^2+^‐dependent inactivation of particular splice variants (Yang *et al*. 2014).

**Figure 3:**
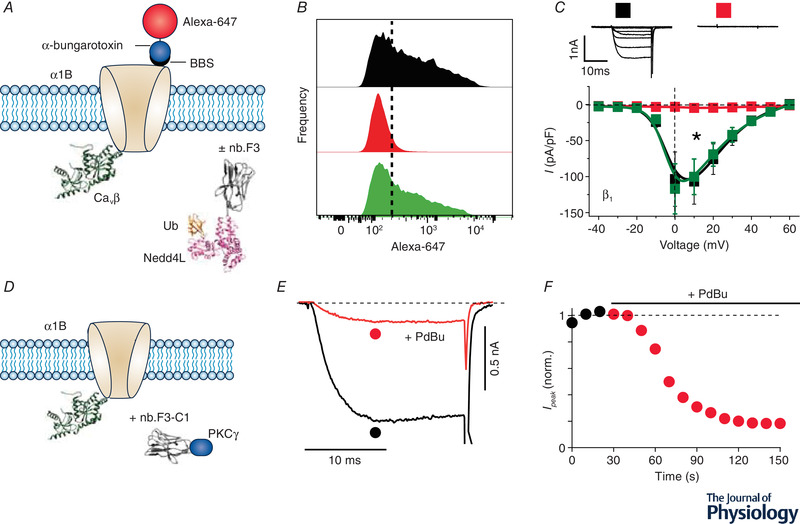
Mimicking RGK‐mediated Ca_V_ inhibition mechanisms with an engineered Ca_V_β‐targeted nanobody *A*, schematic diagram of experimental paradigm. Recombinant Ca_V_2.2 (α_1B_) with an extracellular bungarotoxin‐binding site (BBS) epitope is co‐expressed Ca_V_β without (control) or with a Ca_V_β‐targeting nanobody (nb.F3) fused to catalytic HECT domain of the E3 ubiquitin ligase, NEDD4L. Surface channels are measured by exposing non‐permeabilized transfected cells to Alexa 647‐conjugated bungarotoxin. *B*, histograms of surface BBS‐α_1B_ assessed by flow cytometry in cells expressing no nanobody (top), nb.F3‐NEDD4L (middle), or nb.F3‐NEDD4L^*^, a catalytically dead variant (bottom). Results show a substantial decline in surface density when the channel is co‐expressed with nb.F3‐NEDD4L. *C*, exemplar (top) and population *I‐V* curves (bottom) in cells expressing α_1B_ + β_1b_ alone (■) or with either nb.F3‐NEDD4L (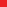) or nb.F3‐NEDD4L^*^ (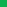) co‐expression. *D*, schematic diagram of experimental paradigm. HEK293 cells are co‐transfected with recombinant Ca_V_2.2 (α_1B _+ β_3_) and nb.F3‐C1_PKCγ_. *E* and *F*, exemplar currents and diary plot showing rapid and deep PdBu‐induced inhibition of Ca_V_2.2 currents in cells expressing α_1B_ + β_3_ + nb.F3‐C1_PKCγ_.

Deployment of genetically encoded Ca_V_ channel inhibitors derived from endogenous proteins (such as Rem_1‐265_‐C1_PKCγ_, β_3_‐C1_PKCγ_, and 14‐3‐3‐C1_PKCγ_) *in vivo* could potentially have unwanted effects owing to over‐expression of these modified natural proteins. As such, we sought to develop genetically encoded Ca_V_ channel inhibitors that would have limited off‐target effects relative to their inhibition of HVA Ca_V_ channels. Given the importance of Ca_V_β‐binding in RGK‐mediated Ca_V_1/Ca_V_2 inhibition, we first isolated nanobodies targeted to auxiliary Ca_V_β subunits. We immunized a llama with purified β_1_ and β_3_ subunits, isolated lymphocytes, amplified nanobodies by PCR, and cloned into a phagemid vector to generate a V_HHS_ phage library. Several nanobody binders to β_1_ were isolated using phage display and an ELISA assay. One of these nanobodies, termed nb.F3, bound all four Ca_V_β isoforms when expressed in cells (Morgenstern *et al*. 2019), which was not surprising given the high homology among these auxiliary subunits in their conserved src homology 3 (SH3) and guanylate kinase (GK) domains (Chen *et al*. 2004; Opatowsky *et al*. 2004; Van Petegem *et al*. 2004). Purified nb.F3 bound Ca_V_β with high affinity (∼12 nM) and 1:1 stoichiometry as assessed by isothermal calorimetry. When expressed with reconstituted Ca_V_2.2 and Ca_V_1.2 channels in HEK293 cells, nb.F3 appeared functionally inert, as it had no impact on channel trafficking to the plasma membrane or on whole‐cell currents. Therefore, nb.F3 provided an ideal Ca_V_β‐targeting module that could potentially be modified to generate a genetically encoded Ca_V_ channel inhibitor exploiting the mechanisms we had identified for RGK proteins. We first sought to mimic the impact of RGKs on decreasing the channel surface density by fusing the catalytic HECT domain of the ubiquitin ligase, Nedd4L, onto nb.F3. The rationale for this approach is that in many ion channels and membrane proteins, ubiquitination typically reduces surface density and, often, enhances protein degradation as well (Abriel & Staub, 2005; Jespersen *et al*. 2007; MacGurn *et al*. 2012; Kanner *et al*. 2017). In heterologous cells, nb.F3‐Nedd4L decreased the surface density of reconstituted Ca_V_2.2 and Ca_V_1.2 channels without enhancing the degradation of the pore‐forming α_1B_ and α_1C_ subunits, respectively (Fig. 3*A* and *B*) (Morgenstern *et al*. 2019). Whole‐cell patch clamp experiments demonstrated that nb.F3‐Nedd4L essentially eliminated reconstituted Ca_V_1.2, Ca_V_1.3 and Ca_V_2.1‐Ca_V_2.3 channel currents (Fig. 3*C*). Therefore, we named nb.F3‐Nedd4L as Ca_V_‐aβlator, reflecting it's exceptional efficacy to inhibit HVA Ca_V_ channels by targeting auxiliary Ca_V_β subunits. Ca_V_‐ablator also proved effective in eliminating endogenous Ca_V_1/Ca_V_2 channels in pancreatic β‐cells, dorsal root ganglion (DRG) neurons and cardiac myocytes (Morgenstern *et al*. 2019). Examination of how Ca_V_‐ablator eliminated Ca_V_1.2 currents in ventricular cardiomyocytes indicated that pore‐forming α_1C_ subunits were re‐directed from dyadic junctions to intracellular compartments, specifically Rab 7‐positive late endosomes (Morgenstern *et al*. 2019).

We have also explored whether we could also use nb.F3 to create a small‐molecule‐inducible genetically encoded Ca_V_ channel inhibitor that exploited the ChIMP mechanism. We generated nb.F3‐ C1_PKCγ_ and co‐expressed it with recombinant Ca_V_1.2 channels. Exposure of cells to phorbol ester resulted in a rapid decline in current that was not observed in control cells lacking nb.F3‐C1_PKCγ_, indicating that nb.F3 permits inducible inhibition of Ca_V_1/Ca_V_2 channels via the ChIMP method (Fig. 3*D–F*).

## Conclusion

In summary, this review highlights work focused on understanding the mechanisms by which RGK proteins potently inhibit Ca_V_1‐ and Ca_V_2‐family channels and exploiting mechanistic insights to create novel genetically encoded Ca_V_ channel inhibitors. This work has led to the development of intracellular acting genetically encoded Ca_V_ channel inhibitors that can be controlled by either small molecules or light, and that have the capacity to block Ca_V_1.2 channels in cardiac myocytes with subcellular specificity. Genetically encoded Ca_V_ channel inhibitors have potential utility as therapeutics for indications such as chronic pain, with the advantage that their expression can be restricted to target tissues or cell types of interest, thereby circumventing off‐target effects. The viability of such gene therapy approaches has been advanced by continually improved development of viral and non‐viral gene delivery methods *in vivo*. For such potential therapeutic applications, it would be important to develop variants whose potency can be controlled either through dosage or with a small molecule. The nanobody‐based approach offers opportunities to design novel genetically encoded Ca_V_ channel inhibitors that can eliminate or modulate Ca_V_ channel complexes on the basis of identity of the associated β subunit isoform. This would be a key enabling tool to probe the potential role of auxiliary β subunits in organizing distinct Ca_V_ channels into distinct signalling complexes that permit functional diversification of Ca^2+^ influx via Ca_V_ channels in individual cells. Finally, some of the approaches described here may be generalizable to develop genetically encoded inhibitors or modulators for other ion channels and membrane proteins. Indeed, we have previously shown that the nanobody‐based targeted ubiquitination approach can be used to inhibit KCNQ1 channels by eliminating them from the cell surface (Kanner *et al*. 2017).

## Additional information

### Competing interests

None declared.

### Funding

This work was supported by grants from the National Institutes of Health (RO1‐GM107585, RO1‐HL121253, and 1RO1‐HL122421) to H.M.C.
